# Do parliaments underrepresent women’s policy preferences? Exploring gender equality in policy congruence in 21 European democracies

**DOI:** 10.1080/13501763.2017.1423104

**Published:** 2018-01-31

**Authors:** Sarah C. Dingler, Corinna Kroeber, Jessica Fortin-Rittberger

**Affiliations:** a Department of political science, University of Salzburg, Salzburg, 5020Austria

**Keywords:** Gender equality, policy preference congruence, substantive representation, women

## Abstract

Although there are considerably more men than women in most parliaments around the world, we know little about whether male-dominated legislatures neglect women’s policy preferences. Our article addresses this gap by analysing the congruence of policy preferences between women, men and their elected representatives. We endeavour to answer two questions. Are women’s policy preferences underrepresented in modern democracies? If so, which factors explain the size of the gender gaps in policy preference congruence? Comparing 21 European countries, we show that women’s preferences actually tend to be more accurately represented in parliaments than those of men. Moreover, our analyses reveal that this unanticipated finding is not driven by the share of female office-holders, but rather by levels of women’s turnout, which leads us to conclude that who votes is more important than who represents for policy preference congruence.

## Introduction

one of the bedrock principles in a democracy is the equal consideration of the preferences and interests of all citizens. (Verba 2003: 663)

Despite their numerical underrepresentation in the parliaments and assemblies in modern democracies, are women’s policy preferences equally well reflected by elected officials compared to those of men? Women tend to care about issues pertaining to reproductive rights, gender equality and other feminist matters that are usually underrepresented in parliaments (Dahlerup 1988; Mansbridge 1999; McAllister and Studlar 1992; Sawer 2012; Schwindt-Bayer and Mishler 2005; Wängnerud and Sundell 2012). Taking aside issues that directly relate to gender, women exhibit more liberal ideological orientations than men (Gidengil *et al*. 2003; Inglehart and Norris 2000), so that gender imbalances in presence could easily translate into poor representation of their preferences. This article examines how the representation of women’s policy preferences differs from those of men across a broad variety of policy fields, as well as the factors shaping this relationship. For this purpose, we compare the level of congruence of policy preferences by exploring the extent to which positions in parliaments correspond to and reflect those of both genders (Monroe 1979; Powell 2000). Since parliaments are lopsided in favour of male representatives, it would be fair to expect that policy preference congruence would also favour male preferences. To date, however, this relationship has received scant empirical verification.

To tackle the issue of substantive representation, this study adopts a multidimensional concept of women’s preferences, covering seven policy areas – views on the free market, welfare state redistribution, the environment, lifestyles, immigration, multiculturalism and religious principles – to capture the diversity of women’s policy positions outside traditional feminist issues. This approach widens the scope of inquiry well beyond existing research, which examines either parliamentary interventions and advocacy on behalf of women (e.g., Dahlerup 2006; Sawer 2012; Schwindt-Bayer and Mishler 2005; Studlar and McAllister 2002; Swers 2002), or congruence between voters and élites on left–right placements (e.g., Bernauer *et al*. 2015; Blais and Bodet 2006; Golder and Stramski 2010; Powell 2000, 2009).

It is widely believed that the gender of elected representatives influences their legislative preferences (e.g., Burrell 1994; Norris and Lovenduski 1995). Hence, equality in numbers between male and of female legislators – gender balanced descriptive representation – is frequently interpreted as a necessary condition for the meaningful representation of their preferences – substantive representation (Phillips 1995). Our analyses, which build on both the European Values Study and the Chapel Hill Expert Survey in 21 European countries, yield unanticipated results. We find no support for the claim that women’s policy preferences should be less accurately represented compared to those of men in male-dominated legislatures. Quite the contrary, congruence of policy preferences is, at times, closest between women and parliamentary representatives. Moreover, other than one would expect, we do not find the highest levels of preference congruence within the group of countries where women assume the largest share of seats. Instead, the most decisive factor for preference congruence we identify is women’s turnout in national elections. In countries where women vote at higher rates than men, elected legislatures mirror women’s policy preferences more closely. Who votes is hence more consequential than who represents for congruence. This finding carries profound implications for research on gender equality in politics, as it suggests that the causal linkage between women’s presence and substantive representation is neither as proximal as previously hypothesized, nor is it circumscribed to specific feminist issues. When we consider a more diverse set of policy preferences transcending typically gendered issues, we find a much higher level of substantive representation than expected, despite comparably low levels of presence.

## Policy preference congruence and women’s representation

The fundamental question of whether women’s policy preferences are underrepresented in modern democracies lies at the core of this article. A vast body of research leads us to expect that the answer is a definite ‘yes’. Assuming that elected officials should respond to the public’s policy preferences within specific areas (e.g., Monroe 1979), the first set of studies addressing this issue considers substantive representation in terms of degrees of congruence in policy preferences between citizens and their representatives. Researchers in this field examine the correspondence between citizens’ preferences and those of parliaments and governments in different institutional settings (e.g., Blais and Bodet 2006; Budge and McDonald 2007; Gilens 2005; Golder and Stramski 2010; Powell 2000; Powell and Vanberg 2000). A related and growing set of literature analyses the representational bias between various sub-populations and parliaments (e.g., Giger *et al*. 2012; Griffin and Newman 2005; Griffin *et al*. 2012) as well as policy outcomes (Branham *et al*. 2017; Enns 2015; Gilens and Page 2014; Rigby and Wright 2013). This research shows that the preferences of historically disadvantaged and excluded groups are least well represented by elected officials and through enacted policies. Contributors identify a general trend of preference underrepresentation of minorities (Griffin and Newman 2007), non-voters (e.g., Campbell 2003; Griffin and Newman 2005; Tate 2003), and relatively poor citizens on the one hand (e.g., Bartels 2008; Branham *et al*. 2017; Ellis 2012; Enns 2015; Giger *et al*. 2012; Gilens 2005; Miller and Stokes 1963; Rigby and Wright 2013), and a preference overrepresentation of economic élites and organized groups in policy outcomes on the other (Gilens and Page 2014). Analysing ideological congruence of women and political parties on a left–right scale, Bernauer *et al*. (2015) provide evidence suggesting that parliaments mirror the ideological positioning of men and women equally well. Yet, the degree to which women are represented in substantive terms hinges on contextual factors: more conservative parties, such as the Republicans in the United States, have been found to better represent policy preferences of men in roll-call voting while Liberal parties, such as the Democrats, tend to better reflect those of women (Griffin *et al*. 2012).

Beyond studies on congruence, the bulk of research addressing factors facilitating women’s substantive representation (e.g., the role of institutional settings or political attitudes) demonstrates that representative bodies are biased towards the preferences of men (McAllister and Studlar 1992; Sawer 2012; Schwindt-Bayer and Mishler 2005; Wängnerud and Sundell 2012). These different studies thus lend support to the expectation that the preferences of women, a traditionally disadvantaged group, should lead to an overrepresentation of men’s preferences.

H_1_: Policy preference congruence between women and parliaments is lower than between men and parliaments.

Moving beyond description, which factors can explain variation in the size of the gender gap in policy preference congruence in a country? The literature provides manifold explanations as to how structural disadvantages outside the legislative arena, for instance lower levels of involvement in non-voting forms of political participation or in organized interest groups, lead to the underrepresentation of women’s preferences (e.g., Kittilson and Schwindt-Bayer 2012; Verba *et al*. 1997). However, for the purpose of this paper, the ensuing section focuses on the most important factors influencing gender equality in substantive representation: presence of women in parliament and turnout.

The ‘politics of presence’ argument by Phillips (1995) suggests that women’s numerical strength in parliaments – descriptive representation – influences the degree to which these elected bodies promote women’s policy preferences. Central to this argument is the idea that the presence of women in parliaments changes the policy-making process. For example, feminist issues are more likely to be brought to the fore as the share of female legislators in elected assemblies increases (Dahlerup 1988; Dovi 2002; Mansbridge 1999; Norris and Lovenduski 1995; Sawer 2012; Schwindt-Bayer and Mishler 2005; Studlar and McAllister 2002; Swers 2002; Wängnerud and Sundell 2012). Moreover, women legislators are more likely to support liberal policies (Burrell 1994), hold liberal attitudes (McAllister and Studlar 1992), and are instrumental in promoting policies that enhance gender quality (Vallance and Davis 1986).

Yet, a number of scholars challenge this straightforward relationship between descriptive and substantive representation. Some argue that critical actors (Chaney 2006; Childs and Krook 2006; Sawer 2012), the positional power of women (Heath *et al*. 2005), party affiliation and ideology (e.g., Griffin *et al*. 2012; McAllister and Studlar 1992), or the broader legislative and societal context (e.g., Chaney 2006; Wängnerud and Sundell 2012) exert a more direct causal impact on the representation of women’s policy preferences. While these studies bring additional nuance to clarify the relationship between descriptive and substantive representation, they do not dispute the underlying rationale that the increasing presence of women in parliaments transforms institutional norms, political discourse and the policy agenda. These considerations inform the second hypothesis of our article:

H_2_: The fewer women elected to parliaments, the lower the policy preference congruence between women and parliaments.

The second potential factor explaining levels of congruence between women and their representatives lies in different propensities to vote. Traditionally, gender gaps in participation were characterized by men turning out to vote in larger proportions than women. Yet, several contributors argue that this gap has been narrowing over time in many countries. In some cases, the gap has even been overturned (Henderson and Jeydel 2010; Inglehart and Norris 2003; Kittilson and Schwindt-Bayer 2012). If men and women display different propensities to engage in political participation, the preferences of the more numerous group at the polls should be more accurately mirrored in parliaments. A vote surplus for one gender should translate into a higher impact on the decision as to which candidates or parties win seats, leaving the policy-making process gendered in favour of the interests and policy preferences of this group. If the proximity of voters to a party’s policy position influences the choice at the ballot box (Downs 1957), sincere voters should support the party whose position is closest to their own. Therefore, if female voters constitute half of the electorate, politicians should be inclined to represent women’s interests.

H_3_: The higher women’s level of turnout, the higher the policy preference congruence between women and parliaments.

## Empirical strategy

Our analyses follow a two-staged strategy to test the three hypotheses elaborated in the previous section. In a first step, we examine whether women’s preferences are underrepresented. In the second step, we investigate which factors explain the size of the gender gaps we uncover. To this end, we draw on data from 21 European countries covering the years from 2005 to 2010.^1^ Our data set encompasses 62 observations – policy areas nested in countries – and is characterized by broad variation in the key variables of interest: the share of female office-holders; the gap in electoral participation between men and women; as well as the gender gap in policy representation.^2^ Since institutional and socioeconomic contexts across the countries in the sample remain comparable, they provide ideal testing grounds for a large-scale comparison and broad generalization potential.

### Measuring the gender gap in policy representation

Previous research on women’s substantive representation has focused on the advancement of feminist issues such as reproductive rights, childcare, access to employment and equal pay, while the bulk of contributions on overall substantive representation has concentrated on citizen–parliament congruence in the left–right ideological dimension. Both approaches are fraught with difficulties. The first cluster of research rests on the problematic assumption that women’s core concerns revolve around a predefined set of policy fields pertaining to their position in society. With the exclusive focus on a unidimensional ideological space, such as the left–right, the second group of contributors also risks oversimplifying policy preferences (Ganghof 2015). Building on the insights from both literatures, yet seeking to overcome some of their limitations, we propose a conceptualization of substantive representation as citizen–parliament congruence in multiple general policy areas in which both men’s and women’s policy preferences actually vary.^3^ With this approach, we avoid making essentializing assumptions about uniform interests for all women in all countries, which many have criticized (e.g., Celis 2006: 88; Celis and Childs 2012: 216; Mackay 2008: 126). Moreover, this strategy allows moving beyond unidimensional ideological arenas.

Measuring the congruence between voters and members of parliament (MPs) requires overlapping data sources for the policy positions of both sets of actors on a series of policy fields. To measure the policy preference distribution of legislators we use the Chapel Hill Expert Survey (CHES) from 2010 (Bakker *et al*. 2015), which assesses the degree to which parties support or oppose a certain policy. We then weight the scores by the parties’ seat share in the previous election. Relying on parties’ policy positions requires us to make the assumption that MPs from the same party share the same ideological preferences (Golder and Stramski 2010: 98). To identify the policy preferences of men and women, we draw on the European Values Study (EVS 2008), which covers most countries included in the CHES (Online Appendix 4 lists all EVS and CHES items). The survey asks respondents to position themselves regarding a series of policies as liberal or conservative. Combining the two data sources allows us to cover seven policy areas for both parties and voters that are standard enough to expect that respondents hold reflected policy preferences and perceive them as salient. These areas encompass policies concerning:

- traditional socioeconomic topics including issue attitudes towards (1) the free market (state regulation or freedom of firms) and (2) redistribution;
- post-materialist value orientations regarding (3) the environment (environmental protection or economic growth) and (4) lifestyles (liberal or conservative positions on homosexuality, abortion, and soft drugs);
- cultural issues covering (5) immigration (restrictive or free movement), (6) multiculturalism (assimilation or diversity) and (7) the role of religious principles in politics (strict separation of religion and politics or guiding function of religion).

To compare men and women’s policy preferences with parliamentary party positions on these seven policy areas, we exploit Golder and Stramski’s (2010) concept of ‘many-to-many’ ideological congruence between a group of citizens and a collective body of representatives. Rather than simply comparing the absolute distance between citizens’ and representatives’ median positions on issues, this approach to measuring congruence compares the entire frequency distributions of citizens’ and MPs’ positions, thereby allowing the most accurate estimate of congruence by considering the variance around the middle position.^4^ For the purpose of this article, we derive our measure for many-to-many congruence by superimposing the frequency distribution of citizens’ answers to the European Value Study policy items and MPs’ positions according to the CHES ranking of their parties.^5^

**Figure 1.**
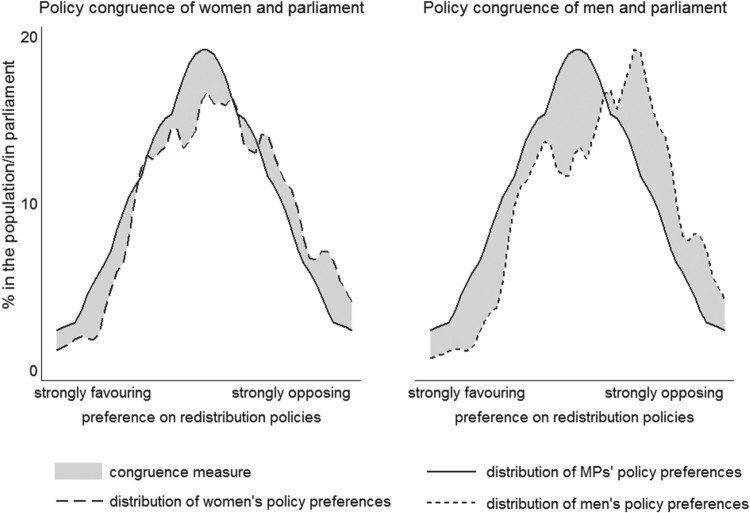
Density function of MPs, women’s and men’s policy preferences on redistribution in the Netherlands. Notes: The figure shows the kernel density function, the frequency is weighted by one hundred in order to provide percentage and simplify interpretation.

Figure 1 illustrates this idea using the example of preferences for redistribution in the Netherlands. The preference distribution of women and MPs is displayed on the left and shows that the parliament is slightly more leftist than female citizens, e.g., representatives are more in favour of redistribution measures than women. Many-to-many congruence of women and parliaments means calculating the size of the grey-shaded area between the distributions of MPs and women. The figure on the right-hand side shows the preference distribution of men and MPs, and many-to-many congruence of men and parliaments. It demonstrates that the policy preferences of men regarding redistribution in the Netherlands are less accurately represented by parliaments than those of women, since the gap between the preference distributions of men and legislators is larger than between those of women and parliaments. If congruence is perfect, there are no differences in the frequency distributions and the congruence measure takes the value 0. The more parliaments and citizens diverge, the larger the measure grows and the lower many-to-many congruence becomes (with the value 2 indicating maximum deviation).

In the first step of our empirical analyses, we compare the means of many-to-many congruence of women and men. In order to obtain a single measure capturing the gender gap for each observation, we further divide the congruence of women and parliaments by the congruence of men and parliaments. This gendered congruence ratio takes the value 1 if the level of congruence of women and parliaments is the same as of men and parliaments. Values smaller than 1 point to a gender gap in which women’s preferences are more accurately reflected in parliaments (as in the situation displayed in the example above), and values larger than 1 indicate an overrepresentation of men’s policy preferences.

### Explanatory variables

The analyses comprise two explanatory variables in order to shed light on the variation in the policy gender gap: women’s descriptive representation and turnout. To test hypothesis 2, we include the proportion of women in the lower house holding office after the last election before 2010 as provided by the Inter-Parliamentary Union (2016).^6^ To measure the gendered differences in turnout and test for the third hypothesis, we analyse self-reported participation according to the EVS.^7^ The survey asks respondents whether they would vote if there was an election the next day. We calculate the share of men and women who report planning to cast a ballot for each country and subtract the share of female from male voters.

### Control variables

Beyond these explanatory variables, we control for numerous factors identified by previous research as affecting policy preference congruence: policy fields, institutional, and socio-economic factors.

#### Policy fields

A dummy variable for each of the seven policy fields takes the value 1 if an observation belongs to a policy field and 0 otherwise. We anticipate the gender gap in policy preferences to vary in size across the seven policy areas, even though the existing literature does not allow us to formulate precise expectations about the direction in which the effects might occur (Gidengil 1995).

#### Institutional factors

First, we control for the proportionality of the electoral system using the mean district magnitude from the Database of Political Institutions (Beck *et al*. 2001), which is calculated as the weighted average of the number of MPs per district. More permissive electoral systems display a higher dispersion of parties with regard to the electorate’s distribution of ideological positions (Dow 2011) and promote higher levels of interest, involvement and participation of women (Kittilson and Schwindt-Bayer 2012). A higher degree of proportionality should hence go hand in hand with a closer reflection of women’s preferences in parliament. Second, we control for ballot structure: a dummy variable takes the value 1 for closed list electoral systems and 0 for open list systems (Beck *et al*. 2001). Closed list systems do not allow voters to punish single representatives, which might lead to women’s underrepresentation in substantive terms (Valdini 2012). Third, as larger parliaments tend to yield more proportional electoral outcomes (Lijphart 1986) and allow for the representation of broader policy preferences, the models include the number of parliamentary seats (Beck *et al*. 2001). Following Salmond (2006), we calculate the logarithm for this variable: increases at low values should make a bigger difference for proportionality than at large values. As a last institutional factor, we include the share of left-wing parties in each parliament (Bakker *et al*. 2015) assuming that left-wing parties present themselves as advocates of disadvantaged groups such as women (Caul 1999; Krook and Childs 2010).

#### Socio-economic factors

Our models include the share of women in the work force (World Bank 2016). Societies with higher rates of women in the work force tend to be more liberal, more open to women’s representation (Inglehart and Norris 2003: 138) and should, thus, better represent women’s preferences. Furthermore, we include a dummy variable that equals 1 if a country belongs to Central and Eastern Europe (0 if otherwise). Owing to their lower democratic experience, these parliaments might be less open to the representation of women’s policy preferences (e.g., Moser 2001). The gender gap should consequently be larger in these countries to the detriment of women.

## The gender gap in policy preference congruence: evidence from 21 European countries

In the first step of our empirical analyses, we explore whether women’s policy preferences are underrepresented in parliaments compared to those of men. Table 1 displays paired *t*-tests for mean comparisons of women-parliament and men-parliament congruence for all observations as well as by policy area.^8^ Contrary to what the bulk of literature leads us to expect, women’s preferences are marginally better represented than those of men: the measure of congruence between men and parliaments is higher than between women and parliaments. Keeping in mind that higher values indicate a lower level of preference congruence, the preferences of men diverge more from those of MPs than those of women. This difference is smaller than zero and reaches conventional levels of statistical significance, which suggests that women’s policy preferences are more accurately mirrored by the elected legislators than those of men. This allows us to reject our first hypothesis, and with it the conventional wisdom, that parliaments discount women’s policy preferences.

**Table 1.** Paired *t*-tests for mean differences between women–parliament and men–parliament congruence for all observations and separately for each policy field.

	Obs.	Women (mean)	Men (mean)	Difference	*p*-value
all	62	1.08	1.13	−0.05	0.00
Free market	11	1.10	1.15	−0.05	0.00
Environment	4	1.44	1.41	0.03	0.18
Immigration	5	1.03	1.05	−0.02	0.36
Multiculturalism	6	1.17	1.16	0.01	0.72
Redistribution	13	1.16	1.20	−0.04	0.04
Religious principle	17	0.93	1.02	−0.09	0.00
Lifestyles	7	1.05	1.10	−0.05	0.20

Turning to policy fields, preferences concerning religious principles, redistribution and free market display a particularly broad gap, reaching high levels of statistical significance despite the low numbers of cases in each category. For example, in Sweden, women’s preferred policies regarding lifestyles (including issues such as homosexual marriage, abortion policies and legalization of soft drugs) are considerably more accurately mirrored in parliament than those of men. More precisely, the congruence measure of women and parliaments is 24 per cent lower than the equivalent for men, which is the largest gender gap in congruence of policy preferences in our sample. We observe the second- and third-largest gaps in policy preference congruence in Lithuania (23 per cent) regarding the question of whether religious principles should guide politicians and in Denmark (21 per cent) concerning the preferred extent of redistribution policies. In all three situations, the congruence measure exhibits a larger deviation between men and parliaments than women and parliaments; in other words, more congruent representation of women’s policy preferences.

It is important to underline that congruence between the two genders and parliaments varies systematically across policy fields. Parliaments reflect men’s preferences for limited environmental protection better than women’s preferences for strong state intervention. MPs also consistently underrepresent women’s preference for openness to a multicultural society. While this suggests the presence of idiosyncratic processes specific to each policy field – the investigation of which lie outside of the scope of the present article owing to limitations in data points – the markers indicate that women’s preferences are more accurately represented in the remaining policy fields.

## Factors determining variation in the gender gap: turnout and descriptive representation

In the second stage of our analysis, we now explain the variation in the size of policy preference congruence between parliaments and the two genders. For this purpose, we use the gendered congruence ratio measure developed in the previous section as a dependent variable for the ensuing analyses. This variable captures the degree of correspondence between women’s policy preferences and parliaments relative to the congruence between men and elected officials. Model 1 in Table 2 displays a baseline ordinary least squares regression model with our core variables of interest to test hypotheses 2 and 3, while the remaining models integrate three sets of control variables.

**Table 2.** Linear regression of the gendered congruence ratio on the share of women in parliament and the gender gap in turnout with different sets of control variables.

	Model 1	Model 2	Model 3	Model 4
	b/se	b/se	b/se	b/se
Main explanatory variables				
% women in parliament	−0.0008 (0.0009)	−0.0010 (0.0008)	−0.0009 (0.0010)	−0.0015 (0.0014)
Gender gap in turnout	−0.0055*** (0.0012)	−0.0037** (0.0017)	−0.0056*** (0.0018)	−0.0058*** (0.0013)
Policy area				
Environment		0.0789*** (0.0203)		
Multiculturalism		0.0692** (0.0328)		
Institutional control variables				
Mean District Magnitude			−0.0001 (0.0001)	
Closed lists			−0.0081 (0.0221)	
Number of parliamentary seats (log)			0.0052 (0.0144)	
% seats for left-wing parties			0.0004 (0.0007)	
Socioeconomic controls				
% women in work force				0.0004 (0.0017)
Central and Eastern European country				−0.0149 (0.0145)
Constant	0.9745*** (0.0196)	0.9693*** (0.0178)	0.9402*** (0.0901)	0.9961*** (0.0315)
Observations	62	62	62	62
*R*^2^	0.084	0.209	0.096	0.088

Notes: All models are simple linear regressions, *p < 0.10, **p < 0.05, ***p < 0.01; standard errors are clustered at the country level.

**Figure 2.**
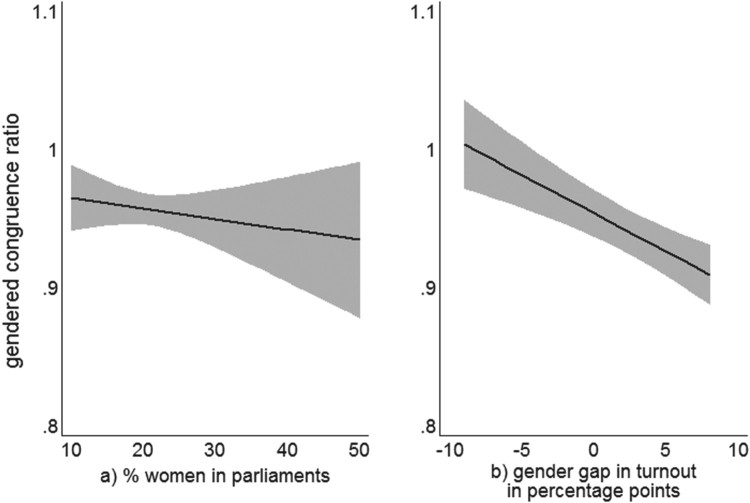
Linear prediction of the gendered congruence ratio for a) the proportion of women in parliaments, and b) the gender gaps in turnout with 95 per cent confidence intervals. Notes: Estimations based on Model 1 in Table 2.

We notice that a higher share of female legislators is associated with only slightly higher levels of congruence between parliaments’ orientations and women’s policy preferences compared to those of men (see Table 2). The coefficient displays a negative sign throughout all models, but the effect is weak and fails to reach conventional levels of statistical significance as visible in Figure 2(a) plotting the marginal effect. Notwithstanding the absence of statistical significance, our models estimate that 10 per cent more female MPs have the effect of decreasing the deviation between women–parliament policy preferences by 0.8 per cent relative to the gap between men and parliaments. In other words, whether a parliament has many or few women affects the predicted congruence ratio only marginally. The ‘politics of presence’ argument, and the corollary hypothesis that unbalanced women’s descriptive representation leads to the overrepresentation of men’s preferences, when operationalized as congruence, receives very limited empirical support.

With regard to hypothesis 3, the gender gap in turnout stands out as the one and only explanatory variable that retains statistical significance at the conventional level throughout all model specifications.^9^ In a nutshell, the more women turn out to vote, the better their preferences are reflected in parliaments. Figure 2(b) displays the marginal effect of gender differences in turnout on the gender gap in preference congruence (based on Model 1). Even if women’s turnout is 9 percentage points lower than that of men – as in the case of Latvia – the model predicts a gendered congruence ratio slightly above 1, which implies that parliaments represent the policy preferences of men and women equally well. As electoral participation of women increases, the ratio falls below 1 and indicates a bias towards the preferences of women. In Portugal, where female electoral participation exceeds male participation by 8 per cent, the congruence measure is 10 per cent higher for men and parliaments than for women and parliaments, meaning that it clearly favours women. In our group of 21 European democracies, higher levels of female turnout compared to that of men lead to more congruent representation of women’s policy preference in parliaments, which is consistent with Griffin and Newman’s (2005) finding that voters are better represented by elected officials than non-voters in the United States.

We ran a series of additional models, introducing control variables, in order to rule out confounding variables as explanations. The parameter estimates for the share of women in parliaments and the gendered differences in turnout (our key variables of interest) remain unchanged by the introduction of any of these control variables, indicating that our results are robust to alternative specifications.^10^

Model 2 incorporates policy preferences in matters of the environment and multiculturalism following the comparison of means presented in Table 1, indicating that women’s preferences are less accurately reflected than men’s in these policy fields. The results of these additional controls suggest that parliaments match men’s policy preferences better than those of women in these fields, while the relationship is reversed for religious principles, deregulation, redistribution, the free market and lifestyles.^11^ Model 3 and 4 integrate a series of country-level variables, yet none of these additional factors exerts a statistically significant effect on the gendered congruence ratio. Even though the institutional setting and the socioeconomic context are the central factors explaining the level of correspondence between all citizens and their elected representatives, as documented in a large literature, these factors lose their explanatory power when we seek to account for differences between genders.

## Conclusions

This article contributes to theoretical and normative debates about the quality of women’s substantive representation in parliaments and the extent to which women’s turnout and descriptive representation help us to understand different patterns of congruence in policy preferences. Contrary to conventional wisdom, which suggests a general underrepresentation of women in terms of substantive representation, we find that parliaments tend to mirror women’s policy preferences slightly more accurately than those of men in the countries we have analysed, with the notable exception of two policy fields, environment and multiculturalism. This finding is at odds with most previous research analysing both the level of correspondence between parliaments and traditionally disadvantaged or excluded groups (Bartels 2008; Ellis 2012; Gilens 2005; Giger *et al*. 2012; Miller and Stokes 1963) and the inclusion of feminist issues on the legislative agenda (McAllister and Studlar 1992; Sawer 2012; Schwindt-Bayer and Mishler 2005; Wängnerud and Sundell 2012). Our analyses validate the notion that there is great diversity in what women expect from elected officials (Beckwith 2014): women’s preferences on mainstream policy issues, on the one hand, and gender-specific topics, on the other, lead to different conclusions about gender equality in representation. While most parliaments might still discount feminist issues for the various reasons explored in the literature, paradoxically, elected officials in Western European countries largely mirror of women’s preferences concerning immigration, religious principles, redistribution, the free market and lifestyles.

We find little support for the ‘politics of presence’ argument. Our analyses show that the share of female office-holders does not have a clear effect on the degree to which parliaments mirror women’s preferences. Although female officeholders have been found to transform institutional norms, political discourse and the policy agenda (Dahlerup 1988; Dovi 2002; Mansbridge 1999; Norris and Lovenduski 1995; Sawer 2012; Schwindt-Bayer and Mishler 2005; Studlar and McAllister 2002; Swers 2002; Wängnerud and Sundell 2012), descriptive representation is not a necessary condition for substantive representation. Our study therefore provides key evidence clarifying the relationship between the different dimensions of representation. If substantive representation is understood as congruence in policy preferences, the causal linkage between descriptive and substantive representation is not as proximal as previously thought, because male and female MPs are equally willing and able to take up women’s heterogeneous policy preferences. For other dimensions of representation, women’s presence in parliaments, as well as their role as critical actors, still carries some weight (e.g., Childs and Krook 2006; Griffin *et al*. 2012; Heath *et al*. 2005). Most importantly, the share of women in elected office can influence other facets of substantive representation, in particular the promotion of women’s issues such as gender equality, maternity, childcare or equal pay. Moreover, the descriptive representation of women in parliaments also generates symbolic representation and promotes justice (Alexander 2012; Mansbridge 1999; Phillips 1995).

While the representation of women’s policy preferences is not a direct function of their numerical strength in parliaments, it is still contingent on another form of presence: women in the electorate and their propensity to turn out to vote. The more women turn out to vote in parliamentary elections, the better parliaments reflect their preferred issue opinions. Only in the few countries where women constitute less than half the electorate, for example Latvia, do we witness a closing of the gender gap in policy preference congruence, with women’s policy preferences being on par with those of men. Women’s higher vote shares make them decisive factors for which party will assume a larger share of the seats. However, the composition of turnout might not only affect the election of representatives, but also policy agendas of parliaments as well as policy content (Lijphart 1997). According to this rationale, vote-maximizing parties should be motivated to closely reflect the policy preferences of the majority of their electorate and thus move the content of their party manifestos closer to voters’ ideal points. Hence, if the electorate is biased in terms of gender, income, age or ethnic background, legislatures might be biased as well as a consequence of shifting policy content concerning substantial issues, such as redistribution towards the preferences of the dominating group. Yet, the question whether electorates influence the preferences of élites or whether the causal arrow points the other way around – that parliaments send cues to the voters, who adapt their preferences according to their policy offers – remains unanswered. To address this question, temporal analyses capturing the extent to which changes in the composition of turnout shape parliaments’ policy orientation offer promising avenues for future research.

## Supplementary Material

RJPP_1423104_Appendix.pdf

## Notes

1. Online Appendix 1 lists all countries and years included in the analyses.

2. Data documentation and summary statistics for all variables are listed in Online Appendices 2 and 3.

3. The inclusion of a policy area for a country in which there is no difference between male and female preferences would lead to an overestimation of equality in representation. We identified cases where policy preferences between men and women differ using *t*-tests (5 per cent level, results in Online Appendix 4). To ascertain that this case selection strategy does not affect results, the main model of this article was estimated using two alternative samples including all observations with a statistically significant gender gap in policy preferences (1) in t-tests (10 per cent level, Online Appendix 7, Test 1) and (2) in Kolmogorov–Smirnov tests (Online Appendix 7, Test 2).

4. To ensure that this operationalization does not drive the findings, we conducted a robustness check (see Online Appendix 7, Test 6) using a difference measure as dependent variable. In addition, we consider alternative survey items as measures for citizens’ policy preferences (see Online Appendix 2 for details and Online Appendix 7, Test 5 for findings).

5. To calculate the congruence measure items, party and voters’ positions must be measured on the same scale. Since CHES does not report integer numbers, we rescaled and rounded party positions of CHES data to adapt them to the EVS’s scale. Details are reported in Online Appendix 2.

6. We conducted two additional robustness tests (Online Appendix 7, Tests 7 and 8) using the share of women in parliaments during the same legislative period as the CHES or the previous legislative period. This different operationalization does not change the findings presented.

7. Following a similar approach as previous studies (e.g., Brady *et al*. 1995; Norris 2003), our analyses only include individuals who report voting intentions. While voting intention is biased upwards in surveys, previous research does not find systematic gender gaps in overreporting of voting (Karp and Brockington 2005).

8. Online Appendix 6 contains country-specific information on men-parliament and women-parliament congruence.

9. When using *Jackknife* standard errors rather than robust standard errors, the level of statistical significance for the parameter estimate of the gender gap in turnout decreases to *p *= 0.015. Online Appendix 7, Test 13 reports the results of this robustness check.

10. We tested for additional variables and combinations of control variables (Online Appendix 7, Tests 9–12), but the robustness tests do not present evidence justifying other models.

11. We estimated separate models with additional value-based policy areas (religious principles, multiculturalism, lifestyles and redistribution) and other relevant areas (environment, free market, immigration), located in Online Appendix 7, Tests 3 and 4. We further provide separate models for all policy areas in Online Appendix 7, Tests 14–20.
