# Supplementary material for: Do parliaments underrepresent women’s policy preferences? Exploring gender equality in policy congruence in 21 European democracies
Source: J Eur Public Policy. 2018 Jan 31;26(2):302–21. doi: 10.1080/13501763.2017.1423104 (PMC6253787; doi:10.1080/13501763.2017.1423104)

Appendixes for: Sarah C. Dingler, Corinna Kroeber, & Jessica Fortin-Rittberger. "Are women's policy preferences underrepresented in parliaments? Exploring gender equality in policy congruence in 24 European democracies."

Appendix 1: List of countries and timing of measurement for key variables.

Country	Abbr.	Timing: EVS	Timing: Composition of parliaments	Timing: Share of women
Austria	AUS	07/08-10/08	2008	2008
Belgium	BE	04/09-08/09	2010	2007
Bulgaria	BUL	04/08-06/08	2009	2009
Czech Republic	CZ	05/08-11/08	2010	2006
Denmark	DK	04/08-09/08	2007	2007
Spain	ESP	05/08-07/08	2008	2008
Estonia	EST	07/08-08/08	2007	2007
Finland	FIN	07/09-07/09	2007	2007
France*	FR	05/08-09/08	2007	2007
Germany	GE	09/08-02/09	2009	2009
Greece	GR	09/08-10/08	2009	2009
Hungary	HUN	11/08-01/09	2010	2006
Ireland*	IRL	06/08-08/08	2007	2007
Italy	IT	10/09-12/09	2008	2008
Latvia	LAT	06/08-10/08	2010	2006
Lithuania	LITH	07/08-09/08	2008	2008
Netherlands	NL	05/08-10/08	2010	2006
Poland	POL	06/08-09/08	2007	2007
Portugal	POR	05/08-08/08	2009	2009
Romania	ROM	04/08-06/08	2008	2008
Slovenia	SLE	03/08-06/08	2008	2008
Slovakia	SLO	07/08-08/08	2010	2006
Sweden	SV	09/09-01/10	2010	2006
United Kingdom*	UK	08/09-03/10	2010	2005

Annotations: Countries marked with a * were only included for the comparison of men's and women's policy preferences, but dropped out at that point due to a lack of statistical significant differences in policy preferences.

Appendix 2: Data documentation

For „Are women's policy preferences underrepresented in parliament? Exploring gender equality in policy congruence in 24 European democracies

The data file contains three data sets: the original data set (62 observations), the extended data set for test 4 (with 72 observations), and alternative indices data set for test 3 (with 62 observations). We observe policy areas clustered in countries from 24 European democracies between 2007 and 2010 in all data sets. While the sample and the operationalization of the gendered congruence ratio vary, all data sets contain the same variables as described below.

cntry

Short description:	Name of country

gendered_congruence

Short description:	Gendered congruence measure
Type:	Continuous, 0 to infinity with<br><1 indicating lower women-parliament congruence than men-parliament congruence ,<br>= 1 indicating equal women-parliament and men-parliament congruence, and<br>>1 indicating higher women-parliament congruence than men-parliament congruence.
Data source(s):	Own calculations based on the European Values Study (2008) and Chapel Hill Expert Survey (Bakker et al. 2010).
Details:	The gendered congruence measure is calculated as follows: $x = \frac{\sum_x \lvert F_{women}(x) - F_{\text{parliament}}(x)\rvert}{\sum_x \lvert F_{men}(x) - F_{\text{parliament}}(x)\rvert}$

women_parliament_congruence

Short description:	Congruence between women's and MPs' policy preferences
Type:	Continuous, 0 to 2 with smaller values indicating higher congruence.
Data source(s):	Own calculations based on the European Values Study

	(2008) and Chapel Hill Expert Survey (Bakker et al. 2010).
Details:	Women-parliament congruence is calculated following Golder and Stramski (2010): $\sum_x \lvert F_{\text{women}}(x) - F_{\text{parliament}}(x) \rvert$ With F_{women} indicting the distribution for women, and $F_{parliament}$ the distribution for parliamentary parties, and x are all values of an item.

men_parliament_congruence

Short description:	Congruence between men's and MPs' policy preferences
Type:	Continuous, 0 to 2 with smaller values indicating higher congruence.
Data source(s):	Own calculations based on the European Values Study (2008) and Chapel Hill Expert Survey (Bakker et al. 2010).
Details:	Women-parliament congruence is calculated following Golder and Stramski (2010): $\sum_x \lvert F_{\text{men}}(x) - F_{\text{parliament}}(x) \rvert$ With F_{men} indicting the distribution for men, and $F_{parliament}$ the distribution for parliamentary parties, and x are all values of an item.

womenproportion

Short description:	Share of female office-holders (in %)
Type:	Continuous, 0 to 100
Data source(s):	Inter-Parliamentary Union (2016), latest election before 2010

Womenproportion_ches

Short description:	Share of female office-holders (in %)
Type:	Continuous, 0 to 100
Data source(s):	Inter-Parliamentary Union (2016), in CHES election year

Womenproportion_pre_ches

Short description:	Share of female office-holders (in %)
Type:	Continuous, 0 to 100
Data source(s):	Inter-Parliamentary Union (2016), in election before the CHES election year

voting_gap

Short description:	Difference between men’s and women’s turnout (in %-points)
Type:	Continuous
Data source(s):	Own calculations based on the European Values Study (2008)
Details:	The variable measures self-reported participation according to the EVS. The survey asks respondents, whether they would vote if there was an election the next day. We calculate the share of men and women that report planning to cast a ballot for each country and subtract the share of female from male voters.

number_parliamentaryseats

Short description:	Number of seats in parliament (total)
Type:	Continuous
Data source(s):	Database of Political Institutions (Beck et al. 2001)

number_parliamentaryseats_log

Short description:	Number of seats in parliament (log)
Type:	Continuous
Data source(s):	Database of Political Institutions (Beck et al. 2001)

district_magnitude

Short description:	Mean district magnitude of the lower house
Type:	Continuous
Data source(s):	Database of Political Institutions (Beck et al. 2001)
Details	In case this detailed information was not available, Beck et al. (2001) divided the number of seats by the number of districts. The Database of Political Institution does not provide any information on Germany. We divided the number of seats (without overhang seats) by the number of districts to be able to include the German case.

closed_list

Short description:	Closed list electoral system
Type:	Binary<br>0 ‘no closed list electoral system’<br>1 ‘closed list electoral system’
Data source(s):	Database of Political Institutions (Beck et al. 2001)

Details	The Database of Political Institutions does not provide information for Germany. We coded Germany as closed lists in accordance with the coding provided by the Comparative Study of Electoral Systems (2016).
disproportionality	
Short description:	Closed list electoral system
Type:	Continuous
Data source(s):	Gallagher Electoral Disproportionality Data (Gandrud 2015)
enp	
Short description:	Effective number of parliamentary parties
Type:	Continuous
Data source(s):	ParlGov (Döring and Manow 2015)
leftwing_strength	
Short description:	% seats for left-wing parties in parliament
Type:	Continuous
Data source(s):	Chapel Hill Expert Survey (Bakker et al. 2010)
rightwing_government	
Short description:	Right-wing government
Type:	Binary<br>0 ‘no right-wing government’<br>1 ‘right-wing government’
Data source(s):	Database of Political Institutions (Beck et al. 2001)
womenlabourforceparticipation	
Short description:	Women’s participation in the labour force (in %)
Type:	Continuous
Data source(s):	World Bank (2016)
cee_country	
Short description:	Country in Central or East Europe
Type:	Binary<br>0 ‘no CEE country’<br>1 ‘CEE country’
immigrate_policy	
Short description:	Policy area: immigration policy
Type:	Binary

0 ‘no’

1 ‘yes’

redistribution

Short description: Policy area: Redistribution

Type: Binary

0 ‘no’

1 ‘yes’

deregulation

Short description: Policy area: free market

Type: Binary

0 ‘no’

1 ‘yes’

religious_principle

Short description: Policy area: religious principle

Type: Binary

0 ‘no’

1 ‘yes’

multiculturalism

Short description: Policy area: Multiculturalism

Type: Binary

0 ‘no’

1 ‘yes’

environment

Short description: Policy area: environment

Type: Binary

0 ‘no’

1 ‘yes’

lifestyles

Short description: Policy area: lifestyles

Type: Binary

0 ‘no’

1 ‘yes’

sd_difference_gender

Short description: Difference of the standard deviation of the preference distribution of men and women

Type:	Continuous
Data source(s):	Own calculations based on the European Values Study (2008)
Details:	Calculated as the SD_{men} minus the SD_{women}. The larger this value, the more are women's policy preferences clustered around the mean value compared to men's policy preferences.

References

Bakker, R., et al. (2010) 'Measuring party positions in europe: The chapel hill expert survey trend file, 1999-2010', *Party Politics* 21(1): 143-52.

Beck, T., Clarke, G., Groff, A., Keefer, P., and Walsh, P. (2001) 'New tools in comparative political economy: The database of political institutions', *World Bank Economic Review* 15(1): 165-76.

Comparative Study of Electoral Systems. 'Cses module 4: 2011-2016', available at http://www.cses.org/datacenter/download.htm (accessed June 2016).

Döring, H. and Manow, P. 'Parlgov database', available at http://www.parlgov.org/static/static-2014/stable/index.html (accessed April 2016).

European Values Study. 'European values study 2008: Integrated dataset (evs 2008). Za4800 data file version 3.0.0', GESIS Data Archive, available at http://www.europeanvaluesstudy.eu/page/data-and-documentation-survey-2008.html (accessed April 2016).

Gandrud, C. 'Gallagher electoral disproportionality data', available at https://github.com/christophergandrud/Disproportionality_Data (accessed April 2016).

Golder, M. and Stramski, J. (2010) 'Ideological congruence and electoral institutions', *American Journal of Political Science* 54(1): 90-106.

Inter-Parliamentary Union. 'Women in national parliaments', available at http://www.ipu.org/wmn-e/arc/classif311209.htm (accessed May 2016).

World Bank. 'Labor force participation rate, female (% of female population ages 15+) (modeled ilo estimate)', available at http://data.worldbank.org/indicator/SL.TLF.CACT.FE.ZS (accessed May 2016).

Appendix 3: Summary statistics for key variables including mean, standard deviation, minimum and maximum.

Variable name	Mean	Std. Dev.	Min.	Max.
gendered_congruence	0.95	0.07	0.76	1.08
women_parliament_congruence	1.08	0.30	0.46	1.81
men_parliament_congruence	1.13	0.28	0.55	1.77
womenproportion	27.09	10.51	10.40	46.40
voting_gap	0.35	3.29	-9.01	7.63
number_parliamentaryseats	245.82	150.68	90.00	630.00
number_parliamentaryseats_log	5.36	0.53	4.50	6.45
district_magnitude	20.05	30.69	1.00	150.00
closed_list	1 ‘yes’	56.45%	0 ‘no’	43.55%
disproportionality	10.00	6.43	1.00	21.00
enp	4.32	1.66	1.70	8.42
leftwing_strength	40.64	9.01	16.90	64.60
rightwing_government	1 ‘yes’	27.42%	0 ‘no’	72.58%
womenlabourforceparticipation	10.19	5.82	1.00	19.00
cee_country	1 ‘yes’	43.55%	0 ‘no’	56.45%
sd_difference_gender	0.02	0.13	-0.18	0.41

Appendix 5: List of CHES (2010) and EVS (2008) variables and observations.

The table below displays the CHES and EVS items used to measure policy congruence. The CHES items ask experts to place party positions on various issues. The EVS items under scrutiny ask respondents for the personal opinions on a broad range of policy issues. For five out of seven policy areas, the EVS provides several variables measuring preferences in the same policy field. We construct additive indices giving equal weight to all items (see below for details). The decision of which items to include might be consequential, therefore we created alternative measures described below. These alternative operationalizations of the dependent variable do not change our findings. Appendix 7, Test 5 reports the results of this robustness check. To receive the same value range for the variables from the two data sets, we changed the direction of some of the EVS variables (so that higher values indicate more liberal policy positions like for the CHES variables) , and scaled all CHES variables according to the EVS variables (since the EVS scales tend to be smaller).

CHES	CHES Description	EVS	EVS Description	Stat Diff (0.05)	Annotations
Free market	Party position on deregulation of markets<br>0 ‘strongly opposes’<br>10 ‘strongly supports’	 <br>v197	*How would you place your views on this scale?*<br>state gives more freedom to control firms more effectively<br>1 ‘state gives freedom to firms’<br>10 ‘state controls firms’	Austria, Belgium, Bulgaria, Czech Republic, Denmark, Estonia, Germany, Italy, Lithuania, Netherlands, Spain	The EVS variable was recoded so that small values indicate regulation as preference.<br><br>The CHES variable was recoded so that it ranges from 1 to 10.
Environment	Party position towards the environment<br>0 ‘strongly supports environmental protection even at the cost of economic growth’<br>10 ‘strongly economic growth even at the cost of environmental protection’	 <br>v301<br><br><br><br>v300<br><br><br><br>v299	*Do you agree or disagree with the following statements:*<br>Environment: if things continue we will experience a catastrophe<br>1 ‘agree strongly’<br>4 ‘disagree strongly’<br>Environment: humans were meant to rule over nature<br>1 ‘agree strongly’<br>4 ‘disagree strongly’<br>Environment: nature is strong	Austria, Estonia, Finland, Greece	We calculated an index with v296, v297, v299, v300, and v301 in which the five variables have equal weight. For that purpose, v301<br><br>The EVS variables v299 and v300 were recoded so that small values indicate acceptance for a liberal lifestyle.<br><br>The CHES variable was recoded so that it ranges from 1 to 4.<br><br>Another variable might measure environmental

		 <br><br><br>v297<br><br><br><br>v296	enough to cope with impacts of industry<br>1 ‘agree strongly’<br>4 ‘disagree strongly’<br>Environment: interference produces disastrous consequences<br>1 ‘agree strongly’<br>4 ‘disagree strongly’<br>Environment: approaching the limit of people<br>1 ‘agree strongly’<br>4 ‘disagree strongly’		preferences, namely v295 (environment: giving part of income), but is weekly correlated with the other variables. In Test 5 (Appendix 3), the environment policy area is based on an index including v295 as well.
Immigration policy	Party position on immigration policy<br>0 ‘fully opposed to a restrictive policy on immigration’<br>10 ‘fully in favor of a restrictive policy on immigration’	 <br>v272<br><br><br><br>v271<br><br><br><br>v270<br><br><br><br>v268	*Where would you place your views on this scale?*<br>Immigrants will become a threat to society<br>1 ‘will become a threat’<br>10 ‘will not become a threat’<br>Immigrants are a strain on welfare system<br>1 ‘are a strain’<br>10 ‘are not a strain’<br>Immigrants increase crime problems<br>1 ‘make it worse’<br>10 ‘do not make it worse’<br>Immigrants take away jobs from [nationality]<br>1 ‘take away’<br>10 ‘do not take away’	Denmark, Finland, Latvia, Lithuania, Sweden	We calculated an index with v268, v270, v271, and v272 in which the three variables have equal weight.<br><br>The EVS variables were recoded so that small values indicate opposition to though migration policies.<br><br>The CHES variable was recoded so that it ranges from 1 to 10.<br><br>Another variable might measure immigration policy preferences, namely v275 (immigrants living in your country: there are too many). This variable is only weekly correlated with the other measures of immigration policy preferences and has a different scaling. In Test 5 (Appendix 3), the immigration policy area is based on an index including v275 as well.
Multiculturalism	Party position on integration of immigrants and asylum seekers (multiculturalism vs. assimilation)<br><br>0 ‘strongly favors multiculturalism’<br>10 ‘strongly favors assimilation’	 <br>v269	*Where would you place your views on this scale?*<br>Immigrants undermine country’s cultural life<br>1 ‘undermine cultural life’<br>10 ‘do not undermine cultural life’	Denmark, Finland, Latvia, Lithuania, Slovenia, Sweden	The EVS variable was recoded so that small values indicate support for multiculturalism.<br><br>The CHES variable was recoded so that it ranges from 1 to 10.<br><br>Another variable might measure preferences about multiculturalism namely v274 (immigrants living in your country: feels like a stranger). Correlation between

					the two variables is week and scaling differs, so that we chose the variable that we found substantially closest to the CHES question. In Test 5 (Appendix 3), the multiculturalism policy area is based on v274 instead of v269 (the scale then ranges from 1 to 5).
Redistribution	Party position on redistribution from the rich to the poor<br>0 ‘strongly favors’<br>10 ‘strongly opposes’	v194	*Where would you place your views on this scale?*<br>individual-state responsibility for providing<br>1 ‘individuals should take more responsibility for providing themselves’<br>10 ‘the state should take more responsibility to ensure that everyone is provided for’	Belgium, Bulgaria, Czech Republic, Denmark, Germany, Italy, Lithuania, Netherlands, Romania, Slovakia, Slovenia, Sweden	The EVS variable was recoded so that small values indicate redistribution as preference.<br><br>The CHES variable was recoded so that it ranges from 1 to 10.<br><br>Another variable might measure preferences about redistribution namely v198 (equalize incomes vs. incentives for individual efforts). However, the correlation between the responses is extremely low which forbids calculating an index. We chose the variable we perceived as most convincing. In Test 5 (Appendix 3), the redistribution policy area is based on v198 instead of v194.
Religious principle	Party position on role of religious principles in politics<br>0 ‘strongly opposes religious principles in politics’<br>10 ‘strongly supports religious principles in politics’	v134	*How much do you agree with the following:*<br>Politicians who do not believe in God are unfit for public office<br>1 ‘agree strongly<br>5 ‘disagree strongly	Austria, Belgium, Bulgaria, Czech Republic, Denmark, Estonia, Germany, Greece, Hungary, Italy, Latvia, Lithuania, Netherlands, Poland, Portugal, Slovakia, Spain	The EVS variable was recoded so that small values indicate opposition to religious principles in politics.<br><br>The CHES variable was recoded so that it ranges from 1 to 5.
Lifestyles	Party position on social lifestyle (e.g. homosexuality)<br>0 ‘strongly supports liberal policies’<br>10 ‘strongly opposes liberal policies’	v240<br><br>v241<br><br>v236	*Do you think the following can always or never be justified?*<br>Do you justify homosexuality<br>1 ‘never’<br>10 ‘always’<br>Do you justify abortion<br>1 ‘never’<br>10 ‘always’<br>Do you justify taking soft drugs<br>1 ‘never’<br>10 ‘always’	Bulgaria, Denmark, Finland, Portugal, Romania, Slovakia, Sweden	We calculated an index with v240, v241, and v236 in which the three variables have equal weight.<br><br>The EVS variables were recoded so that small values indicate acceptance for a liberal lifestyle.<br><br>The CHES variable was recoded so that it ranges from 1 to 10.<br><br>Further variables might measure lifestyle preferences such as agreement to the statements in v154 (homosexual couples should be able to adopt children)

		and v155 (It is alright to live together without getting married). However, they have different scale for coding which makes it complicated to include them into the index. In Test 5 (Appendix 3), the lifestyle policy area is based on an index combining all 5 variables.

Appendix 4: T-Tests for mean comparison of policy preferences of men and women in seven policy areas.

			Men		Women	
Policy Area	Country	N	Mean	SE	Mean	SE
Redistribution	AUS	1,459	6.885	(0.100)	6.785	(0.091)
	BE*	1,474	6.130	(0.089)	5.778	(0.093)
	BUL*	1,446	6.092	(0.120)	5.642	(0.104)
	CZ*	1,763	6.467	(0.092)	6.058	(0.084)
	DK*	1,464	6.456	(0.080)	6.099	(0.083)
	ESP	1,302	5.430	(0.096)	5.328	(0.083)
	EST	1,409	6.153	(0.122)	5.984	(0.085)
	FIN	1,08	6.515	(0.112)	6.353	(0.140)
	FRA	1,461	6.515	(0.096)	6.263	(0.087)
	GE*	2,029	7.255	(0.083)	7.001	(0.093)
	GR	1,436	5.517	(0.115)	5.488	(0.101)
	HUN	1,497	5.656	(0.101)	5.471	(0.092)
	IRL	971	6.934	(0.134)	6.665	(0.112)
	IT*	1,464	5.508	(0.104)	5.030	(0.093)
	LAT	1,245	5.584	(0.124)	5.326	(0.091)
	LITH*	1,472	6.331	(0.097)	5.998	(0.089)
	NL*	1,542	6.472	(0.083)	5.957	(0.084)
	POL	1,486	5.789	(0.099)	5.657	(0.090)
	POR	1,456	6.586	(0.112)	6.510	(0.105)
	ROM*	1,445	7.295	(0.125)	6.944	(0.109)
	SLE*	1,347	6.063	(0.115)	5.617	(0.104)
	SLO*	1,45	6.576	(0.118)	6.200	(0.096)
	SV*	1,131	7.030	(0.105)	6.549	(0.097)
	UK	1,522	7.254	(0.092)	7.124	(0.079)
Free market	AUS*	1,425	6.463	(0.104)	6.161	(0.094)
	BE*	1,453	5.210	(0.093)	4.939	(0.086)
	BUL*	1,401	5.220	(0.133)	4.812	(0.113)
	CZ*	1,707	5.763	(0.093)	5.457	(0.087)
	DK*	1,419	6.223	(0.085)	5.829	(0.079)
	ESP*	1,269	4.980	(0.098)	5.281	(0.083)
	EST*	1,369	5.494	(0.120)	4.997	(0.083)
	FIN	1,044	5.654	(0.104)	5.584	(0.104)
	FRA	1,457	5.489	(0.107)	5.348	(0.088)
	GE*	1,982	6.260	(0.098)	5.933	(0.092)
	GR	1,413	4.493	(0.120)	4.478	(0.101)
	HUN	1,478	4.365	(0.106)	4.444	(0.099)
	IRL	874	6.125	(0.133)	6.318	(0.105)
	IT*	1,375	5.288	(0.114)	4.878	(0.103)

	LAT	1,187	5.029	(0.119)	4.752	(0.090)
	LITH*	1,333	5.693	(0.108)	5.372	(0.095)
	NL*	1,493	6.010	(0.088)	5.565	(0.074)
	POL	1,425	5.899	(0.113)	5.636	(0.096)
	POR	1,316	5.966	(0.122)	5.800	(0.107)
	ROM	1,349	5.119	(0.137)	4.966	(0.120)
	SLE	1,293	5.760	(0.123)	5.589	(0.104)
	SLO	1,404	5.072	(0.130)	4.768	(0.104)
	SV	1,059	6.581	(0.108)	6.394	(0.100)
	UK	1,448	6.367	(0.097)	6.321	(0.072)
Multiculturalism	AUS	1,461	6.425	(0.114)	6.505	(0.101)
	BE	1,473	5.657	(0.106)	5.825	(0.100)
	BUL	1,295	4.116	(0.129)	4.070	(0.111)
	CZ	1,754	5.385	(0.098)	5.323	(0.091)
	DK*	1,452	4.607	(0.103)	4.320	(0.102)
	ESP	1,317	4.991	(0.113)	5.034	(0.096)
	EST	1,379	5.303	(0.133)	5.267	(0.096)
	FIN*	1,09	4.255	(0.130)	3.747	(0.154)
	FRAU	1,459	5.000	(0.111)	4.945	(0.103)
	GE	1,997	6.254	(0.103)	6.014	(0.104)
	GR	1,422	5.426	(0.126)	5.657	(0.104)
	HUN	1,492	4.925	(0.114)	5.002	(0.110)
	IRL	954	5.818	(0.153)	5.982	(0.115)
	IT	1,433	4.988	(0.114)	4.885	(0.108)
	LAT*	1,208	5.929	(0.129)	5.477	(0.103)
	LITH*	1,329	5.518	(0.108)	5.204	(0.100)
	NL	1,523	5.146	(0.098)	5.172	(0.096)
	POL	1,474	4.270	(0.104)	4.435	(0.098)
	POR	1,411	4.658	(0.119)	4.573	(0.117)
	ROM	1,377	4.799	(0.130)	4.504	(0.115)
	SLE	1,329	5.659	(0.120)	5.169	(0.108)
	SLO*	1,416	4.590	(0.137)	4.485	(0.113)
	SV*	1,063	4.861	(0.144)	3.868	(0.118)
	UK	1,492	6.381	(0.112)	6.415	(0.094)
Immigration	AUS	1,445	6.952	(0.095)	7.108	(0.084)
	BE	1,455	6.370	(0.082)	6.513	(0.078)
	BUL	1,045	6.044	(0.110)	5.882	(0.102)
	CZ	1,65	6.667	(0.078)	6.661	(0.075)
	DK*	1,413	5.628	(0.070)	5.346	(0.079)
	ESP	1,24	5.702	(0.092)	5.868	(0.077)
	EST	1,292	6.449	(0.102)	6.370	(0.075)
	FIN*	1,019	6.067	(0.104)	5.635	(0.125)
	FRAU	1,439	5.334	(0.092)	5.252	(0.084)

	GE	1,966	6.769	(0.087)	6.723	(0.089)
	GR	1,392	6.760	(0.093)	6.899	(0.081)
	HUN	1,45	7.133	(0.075)	7.057	(0.076)
	IRL	916	6.875	(0.111)	6.847	(0.090)
	IT	1,279	6.027	(0.096)	6.093	(0.095)
	LAT*	1,099	6.817	(0.090)	6.530	(0.079)
	LITH*	1,112	6.879	(0.085)	6.648	(0.080)
	NL	1,457	5.817	(0.075)	5.763	(0.077)
	POL	1,349	5.558	(0.085)	5.498	(0.080)
	POR	1,285	5.900	(0.097)	5.959	(0.096)
	ROM	1,197	5.092	(0.104)	4.958	(0.093)
	SLE	1,234	6.436	(0.093)	6.205	(0.091)
	SLO	1,279	6.565	(0.100)	6.632	(0.087)
	SV*	970	5.454	(0.130)	4.644	(0.119)
	UK	1,428	6.983	(0.096)	6.945	(0.080)
Religious principles	AUS*	1,421	2.331	(0.052)	2.525	(0.046)
	BE*	1,464	1.548	(0.037)	1.653	(0.038)
	BUL*	1,302	2.866	(0.049)	2.994	(0.043)
	CZ*	1,7	2.032	(0.037)	2.207	(0.036)
	DK*	1,461	1.408	(0.028)	1.504	(0.030)
	ESP*	1,331	1.981	(0.041)	2.268	(0.041)
	EST*	1,358	2.259	(0.047)	2.479	(0.037)
	FIN	1,066	2.015	(0.050)	2.105	(0.060)
	FRAU	1,461	1.634	(0.042)	1.707	(0.039)
	GE*	1,957	2.210	(0.046)	2.493	(0.051)
	GR*	1,407	2.849	(0.055)	3.059	(0.045)
	HUN*	1,49	2.193	(0.043)	2.476	(0.046)
	IRL	913	2.232	(0.061)	2.354	(0.050)
	IT*	1,419	2.292	(0.044)	2.450	(0.046)
	LAT*	1,196	2.417	(0.047)	2.567	(0.038)
	LITH*	1,364	2.422	(0.038)	2.676	(0.038)
	NL*	1,523	1.590	(0.033)	1.696	(0.034)
	POL*	1,454	2.347	(0.039)	2.580	(0.037)
	POR*	1,397	2.133	(0.051)	2.407	(0.048)
	ROM	1,368	3.422	(0.049)	3.533	(0.040)
	SLE*	1,315	2.041	(0.042)	2.048	(0.037)
	SLO	1,381	1.984	(0.052)	2.221	(0.045)
	SV	1,065	1.614	(0.043)	1.711	(0.041)
	UK	1,476	2.098	(0.042)	2.126	(0.031)
Environment	AUS*	1,185	2.019	(0.026)	1.907	(0.024)
	BE	1,356	2.047	(0.022)	2.023	(0.021)
	BUL	1,1	2.055	(0.028)	1.970	(0.020)
	CZ	1,286	2.193	(0.023)	2.138	(0.025)

	DK	1,069	2.031	(0.025)	2.038	(0.024)
	ESP	924	1.951	(0.026)	1.912	(0.023)
	EST*	1,353	1.925	(0.022)	1.921	(0.022)
	FIN*	820	1.816	(0.033)	1.694	(0.032)
	FRAU	1,337	1.873	(0.021)	1.875	(0.019)
	GE	1,518	1.995	(0.025)	1.949	(0.025)
	GR*	1,148	1.862	(0.021)	1.802	(0.019)
	HUN	807	2.071	(0.032)	2.071	(0.022)
	IRL	559	2.220	(0.038)	2.132	(0.035)
	IT	1,006	2.060	(0.021)	2.017	(0.020)
	LAT	734	2.079	(0.026)	2.062	(0.025)
	LITH	1,062	2.299	(0.028)	2.267	(0.024)
	NL	1,185	2.222	(0.023)	2.198	(0.022)
	POL	901	2.222	(0.033)	2.242	(0.026)
	POR	1,012	2.002	(0.030)	1.974	(0.029)
	ROM	993	1.997	(0.031)	1.993	(0.030)
	SLO	1,101	2.085	(0.020)	2.063	(0.017)
	SV	757	1.979	(0.030)	2.008	(0.025)
	UK	1,047	2.060	(0.025)	2.062	(0.021)
Lifestyles	AUS	1,366	7.030	(0.090)	6.949	(0.078)
	BE	1,456	6.735	(0.079)	6.729	(0.072)
	BUL*	1,259	8.230	(0.071)	7.941	(0.061)
	CZ	1,661	6.748	(0.084)	6.865	(0.078)
	DK*	1,434	5.348	(0.083)	5.031	(0.079)
	ESP	1,242	6.323	(0.108)	6.349	(0.089)
	EST	1,32	8.311	(0.076)	8.285	(0.051)
	FIN*	1,04	6.199	(0.114)	5.691	(0.114)
	FRAU	1,426	6.470	(0.083)	6.282	(0.077)
	GE	1,947	6.837	(0.087)	6.849	(0.080)
	GR	1,371	7.605	(0.093)	7.696	(0.074)
	HUN	1,465	7.859	(0.070)	7.885	(0.066)
	IRL	889	7.436	(0.112)	7.346	(0.099)
	LAT	1,137	8.399	(0.074)	8.390	(0.058)
	LITH	1,227	8.332	(0.067)	8.451	(0.055)
	NL	1,504	5.642	(0.098)	5.767	(0.085)
	POL	1,412	8.219	(0.080)	8.358	(0.069)
	POR*	1,305	7.470	(0.100)	7.169	(0.093)
	ROM*	1,351	8.328	(0.086)	8.637	(0.057)
	SLE*	1,299	7.150	(0.095)	6.911	(0.089)
	SLO	1,268	6.718	(0.108)	7.048	(0.094)
	SV*	1,012	5.155	(0.097)	4.798	(0.080)
	UK	1,451	6.830	(0.096)	6.618	(0.078)

Annotations: The table displays mean comparisons for two-sample t-Tests. Observations marked with a * indicate that the difference in policy preferences between men and women is statistically significant at the five-percent level, so that the observations was included in the analyses.

Appendix 6: Figure of men-parliament and women-parliament congruence by country and policy area.

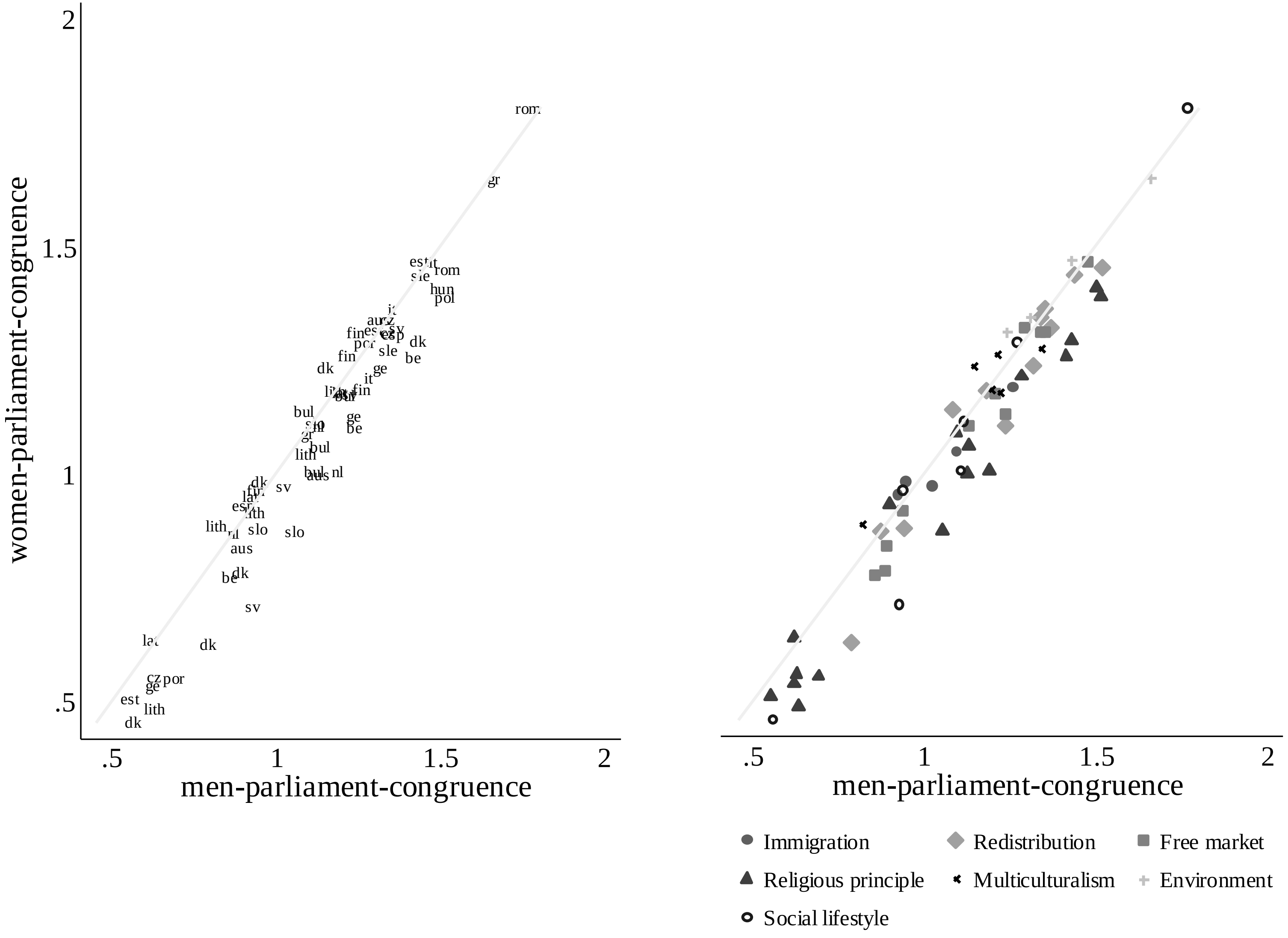

Supplement: RJPP_1423104_Appendix.pdf [file RJPP_A_1423104_SM5053.pdf]
